# IRX-2, a Novel Immunotherapeutic, Enhances Functions of Human Dendritic Cells

**DOI:** 10.1371/journal.pone.0047234

**Published:** 2013-02-07

**Authors:** Bastian Schilling, Malgorzata Harasymczuk, Patrick Schuler, James Egan, Soldano Ferrone, Theresa L. Whiteside

**Affiliations:** 1 University of Pittsburgh, Department of Pathology and University of Pittsburgh Cancer Institute, Pittsburgh, Pennsylvania, United States of America; 2 IRX Therapeutic Inc., Farmingdale, New York, United States of America; New York University, United States of America

## Abstract

**Background:**

In a recent phase II clinical trial for HNSCC patients, IRX-2, a cell-derived biologic, promoted T-cell infiltration into the tumor and prolonged overall survival. Mechanisms responsible for these IRX-2-mediated effects are unknown. We hypothesized that IRX-2 enhanced tumor antigen-(TA)-specific immunity by up-regulating functions of dendritic cells (DC).

**Methodology/Principal Findings:**

Monocyte-derived DC obtained from 18 HNSCC patients and 12 healthy donors were matured using IRX-2 or a mix of TNF-α, IL-1β and IL-6 (“conv. mix”). Multicolor flow cytometry was used to study the DC phenotype and antigen processing machinery (APM) component expression. ELISPOT and cytotoxicity assays were used to evaluate tumor-reactive cytotoxic T lymphocytes (CTL). IL-12p70 and IL-10 production by DC was measured by Luminex® and DC migration toward CCL21 was tested in transwell migration assays. IRX-2-matured DC functions were compared with those of conv. mix-matured DC. IRX-2-matured DC expressed higher levels (p<0.05) of CD11c, CD40, CCR7 as well as LMP2, TAP1, TAP2 and tapasin than conv. mix-matured DC. IRX-2-matured DC migrated significantly better towards CCL21, produced more IL-12p70 and had a higher IL12p70/IL-10 ratio than conv. mix-matured DC (p<0.05 for all). IRX-2-matured DC carried a higher density of tumor antigen-derived peptides, and CTL primed with these DC mediated higher cytotoxicity against tumor targets (p<0.05) compared to the conv. mix-matured DC.

**Conclusion:**

Excellent ability of IRX-2 to induce *ex vivo* DC maturation in HNSCC patients explains, in part, its clinical benefits and emphasizes its utility in *ex vivo* maturation of DC generated for therapy.

## Introduction

Dendritc cells (DC) are specialized, highly potent antigen presenting cells (APC) that are capable of inducing primary immune responses *in vivo* [1]. In cancer patients, the DC frequency and functions are decreased, and these defects account, at least in part, for suppression of tumor antigen (TA)-specific immune responses seen in these patients [2]. DC functions in cancer patients seem to be impaired in multiple ways [2]. DC showing an immature phenotype (iDC) with reduced abilities to prime T cell responses were present in patients with colorectal and breast cancer [3], [4]. In HNSCC patients, accumulations of these iDC correlated with a poor prognosis [5], [6]. These iDC express low levels of co-stimulatory molecules, and thus may not be able to provide signal 2 necessary for priming of T-cell responses. However, little attention has been paid to the ability of DC from cancer patients to process and present TA, a process involving the components of the antigen processing machinery (APM), which is required for the delivery of signal 1 in the induction of primary T-cell responses.

The APM consists of several intracellular proteins responsible for processing, transport and chaperoning of peptides derived mostly, but not exclusively, from endogenous proteins for cross-presentation. After cleavage of these proteins by the proteasome subunits, LMP-2 and LMP-7, the subunits of the transporter associated with antigen processing (TAP), TAP1 and TAP2, transport peptides into the endoplasmic reticulum (ER) [7]. TAP1/TAP2 complexes are then brought into contact with β2-microglobulin (β2m)-HLA class I heavy chain complexes by tapasin [7]. Before trimeric HLA class I heavy chain-β2m-peptide complexes are transported to the cell surface, proper folding catalyzed by the chaperone molecules, BiP, calnexin, calreticulin, and ERp57, takes place in the ER [8]. HLA class I peptide complexes on the cell surface of APC are recognized by CD8^+^ T lymphocytes bearing cognate T cell receptors [8]. Recent studies suggest that up-regulation of the APM component expression correlates with the improved ability of DC to cross-present antigens and to cross-prime cytolytic T lymphocytes (CTL) [9], [10]. Yet, APM component expression and its contribution to DC function in cancer patients have been evaluated only to a limited extent.

Impaired DC functions observed in cancer patients could potentially contribute to tumor escape by negatively regulating anti-tumor T cells [2]. Thus, it would be desirable to correct DC impairments and restore anti-tumor activity of T cells *in vivo*. Systemic delivery of cytokines, e.g., GM-CSF or IFN-α_2b_ to patients with cancer is aimed at the restoration of DC functions and the generation of more robust anti-tumor T-cell responses [11], [12]. Therefore, IRX-2, a cell-derived biologic containing a well-defined mix of cytokines, was recently administered to the HNSCC patients enrolled in a phase II clinical trial. IRX-2 was injected locoregionally in the adjuvant setting with an expectation that it might enhance DC function *in vivo* [13]. The results showed a significant infiltration of tumors with activated T cells after IRX-2 therapy which was associated with prolonged overall survival (OS) [14]. We have previously reported that IRX-2 is able to up-regulate HLA-DR, CD86, CD40 and CCR7 expression and induce IL12p70 production, a cytokine necessary for Th1 polarization, in monocyte-derived DC generated from PBMC of healthy donors (HD) [15]. Although, we attributed the observed positive correlation between T-cell infiltration and OS to improved functions of DC after IRX-2 delivery, no information is available about the mechanisms through which the treatment of DC with IRX-2 might up-regulate T-cell anti-tumor activity. Here, we evaluate *in vitro* effects of IRX-2 on DC and, specifically, on the APM component expression in these cells which determines their potential to present TA to T cells. Our data show that IRX-2 not only enhances functions in mDC obtained from cancer patients and HD, but that it does so more efficiently than the conventional mix of IL-6, IL-1 and TNF-α broadly used for DC maturation. Thus, IRX-2 might be potentially beneficial as an immune therapeutic and a maturation biologic for the production of therapeutic DC.

## Results

### Purity and Phenotype of iDC of Cancer Patients and HD

The purity of iDC from patients and HD was evaluated by microscopic cell counts (morphology) and by flow cytometry (FS/SS properties). DC preparations routinely contained ≥80% of cells with DC morphology, and cell viability routinely exceeded 90% as determined by a trypan blue exclusion test. Table S1, shows that the phenotype of iDC generated from monocytes obtained from HD and HNSCC are not different. However, as shown in Figure S1A and S1B, intracytoplasmic staining of iDC for various APM components revealed a significantly lower expression (p<0.01) of TAP1 and TAP2 in iDC of HNSCC patients relative to that in iDC of HD. The differences were selective since expression of LMP2, Tapasin and Calreticulin was not significantly different in iDC of HNSCC patients as compared to iDC of HD.

### Distinct Phenotype of DC Matured by IRX-2 vs. a Conventional Maturation Cocktail

**Figure 1:**
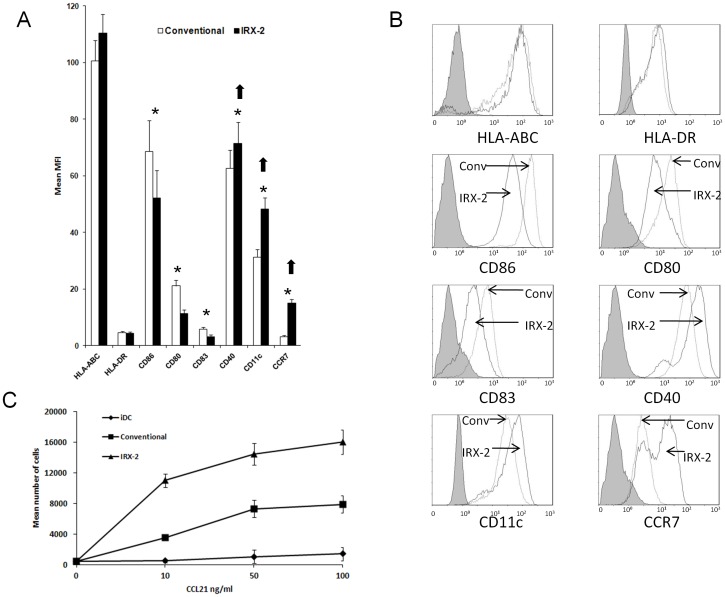
Phenotype and migration of DC matured in IRX-2 or conventional cytokines. (**A**) DC obtained from HNSCC patients were matured for 48 h either with IRX-2 or the conventional maturation cocktail. While conventionally matured DC (white bars) expressed higher levels of CD80, CD83 and CD86 (*, p<0.05), IRX-2 matured DC (black bars) showed higher expression of CD11c, CD40 and CCR7 (*, p<0.05). The data are mean x-fold of MFI ± SEM for cells obtained from 12 different HNSCC patients. (**B**) Representative histograms showing expression of DC markers after maturation with IRX-2 or the conventional cytokine cocktail in DC generated from monocytes of one HNSCC patient. The shaded peaks represent isotype controls. (**C**) Migration of mDC *in vitro*: Migration assays were performed as described in Materials & Methods using DC generated from peripheral blood monocytes of HNSCC patients. While iDC showed very little migration in response to CCL21, both conventional- and IRX-2-matured DC migrated significantly better. Results are shown as the mean absolute numbers of migrated cells ± SEM obtained from 5 different HNSCC patients.

A widely used conventional combination of cytokines for DC maturation consists of TNF-α, IL-1β and IL-6. We compared it with IRX-2 after 48 h of maturation, which results in maximal effects as determined in preliminary studies (data not shown). Both procedures resulted in a significant upregulation of all DC surface markers, including the maturation markers CCR7, CD80 and CD83. However, several differences were observed in the phenotype of mDC derived from monocytes of the same patients but matured either conventionally or by IRX-2 as shown in Figure 1. The conventionally matured mDC had higher expression of CD80, CD83 (p<0.01) and CD86 (p<0.05) than the IRX-2-matured DC. On the other hand, the IRX-2-matured DC expressed significantly higher levels of CCR7 (p<0.01), CD11c (p<0.01) and CD40 (p<0.05) than conventionally matured mDC. As shown in Figure 1, total MHC-Class I and HLA-DR molecules were up-regulated to a similar extent in DC matured with IRX-2 and conventional cytokines. Similar results were obtained when using DCs from HD (data not shown).

### IRX-2-matured DC Produce Higher Levels of IL-12p70 than Conventionally-matured DC

IL-12p70 production by DCs and the IL-12p70/IL-10 ratio have been used as surrogate markers to predict the *in vivo* potency of mDC. Therefore, we tested iDC, IRX-2-matued and conventionally matured DC for their ability to produce IL-12p70 and IL-10. In iDC supernatants, IL-12p70 or IL-10 were not detected (data not shown). Upon maturation in the conventional cocktail or in IRX-2, DC produced detectable levels of both IL-12p70 and IL-10 (Table 1). However, IRX-2-matured DC produced higher levels (p<0.05) of IL-12p70 and lower levels of IL-10 (p = 0.071) than those matured with conventional cytokines. As shown in Table 1, the IL-12p70/IL-10 ratio was significantly greater in the supernatant of IRX-2-matured DC (2.7 vs. 1.4, p<0.05). Interestingly, we observed that DC of HD secreted higher total levels of IL-12p70 (p<0.01) as well as IL-10 than those of HNSCC patients, while the IL-12p70/IL-10 ratios were similar to those seen in HNSCC patients for both maturation cocktails (Tab. 2, 3.0 for IRX-2 and 1.8 for the conventional cocktail).

**Table 1:** IL-12p70 and IL-10 secretion by moDC of HD and HNSCC patients matured with IRX-2 or the conventional maturation cocktail.

Cytokine	HD		p-value
	Conventional (meanpg/ml/10^5^ cells ±SEM)	IRX-2 (meanpg/ml/10^5^ cells ±SEM)	
IL-12p70	25.4±5.9	40.3±7.4	<0.05
IL-10	20.8±4.8	14.4±4.4	0.071
Ratio of mean IL-12p70/IL-10	1.4	2.7	<0.05
	**HNSCC**		
IL-12p70	11.9±1.5	22.6±6.6	<0.01
IL-10	8.1±4.3	6.4±2.9	0.24
Ratio of mean IL-12p70/IL-10	1.8	3	<0.05

**Table 2:** Clinicopathologic characteristics of patients with HNSCC who donated blood for this study.

Age (Range in years)	39–78
Sex	
Male	13
Female	5
Total	18
Tumor Stage	
T_1_	4
T_2_	10
T_3_	1
T_4_	3
Nodal Status	
N_1_	1
N_2_	4
N_3_	0

### Higher Numbers of IRX-2-matured than Conventionally-matured DC Migrate Towards CCL21

To determine the functional significance of a higher percentage of CCR7^+^ cells present in IRX-2-matured than conventionally-matured DC, we tested the ability of DC to migrate towards CCL21. In a transwell migration assay, mDC of HNSCC patients generated in the presences of IRX-2 had a greater capability to migrate (p<0.01) than iDC or mDC exposed to the conventional cytokine cocktail. As shown in Figure 1C, iDC showed very little migration towards CCL21, while IRX-2-induced mDC of the same donors migrated considerably better. In turn, mDC generated in the conventional cytokine mixture migrated less efficiently (mean cells 16,000 vs. 7900, p<0.01).

#### *APM component expression is higher in IRX-2-matured than conventionally-matured DC*

**Figure 2:**
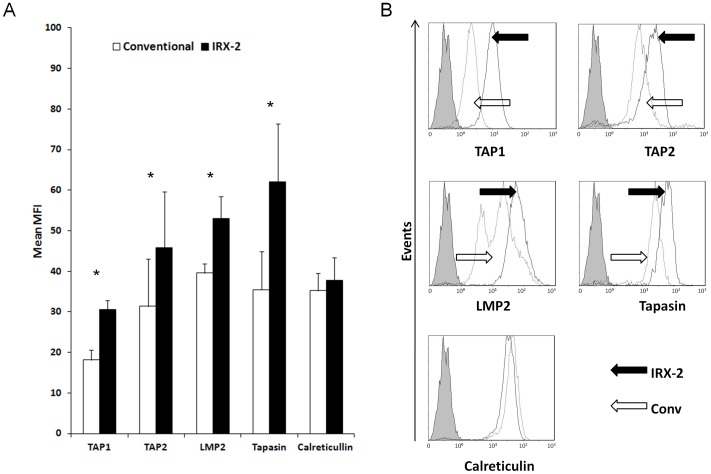
APM expression in mDC. moDC from HNSCC patients were matured for 48 h either with IRX-2 or the conventional maturation cocktail. (A) IRX-2-matured DC (black bars) expressed significantly higher levels of TAP1, TAP2, LMP2 and Tapasin than conventional DC (white bars, *, p<0.05). APM expression was determined by flow cytometry. The data are mean x-fold of MFI ± SEM for cells obtained from 12 different HNSCC patients.

Next, the intracellular expression of APM components in DC matured conventionally or with IRX-2 was compared. Both the conventional cytokine cocktail and IRX-2 up-regulated the expression levels of the APM components LMP2, TAP1, TAP2, Tapasin and Calreticulin as compared to iDC from the same donors (the data for iDC are shown in Figure S1). However, as shown in Figure 2, IRX-2 induced higher levels of LMP2, TAP1, TAP2 and Tapasin (p<0.05 for all) in mDC than did conventional cytokines. No significant differences in the expression of Calreticulin and surface MHC Class I molecules were evident between mDC matured with IRX-2 and the conventional mix.

### IRX-2-matured DC Induce TA-specific CTL in vitro

**Figure 3:**
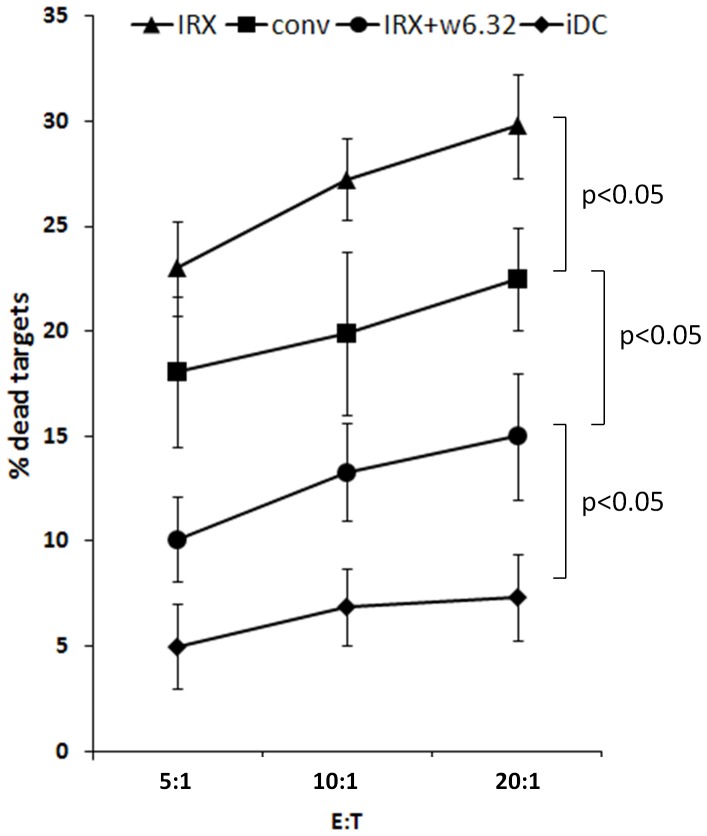
Cytotoxicity of CTL generated in IVS cultures. CTL were induced and expanded using iDC and DC matured either with IRX-2 or the conventional cocktail from HLA-A2^+^ HNSCC patients. CTL primed by conventionally-matured or IRX-2-matured DC were more effective than CTL primed with iDC. CTL generated using IRX-2-matured DC showed higher cytotoxicity than those primed with conventional DC. Blocking MHC-class-I recognition with the mAb w6.32 abrogated cytotoxicity. The data are mean percentages ± SEM of specific killing at different E:T ratios obtained from 4 independent experiments.

The induction of TA-specific T cells (CTL) is the final and critical endpoint of antigen presentation by mDC. In IVS cultures, we generated CTL from PBMC of HLA-A2^+^ HNSCC patients using mDC which were cultured in the presence of IRX-2 or conventional cytokines. Lysates of the HLA-A2^+^ HNSCC cell line PCI-13 served as an antigen source in the IVS culture. As shown in Figure 3, both conventional and IRX-2 matured DC induced CTL which were able to kill PCI-13 target cells. Anti-HLA class I blocking Abs inhibited cytotoxicity and CTL showed only low cytotoxicity against the irrelevant target MCF-7 (data not shown). However, CTL generated in the presence of IRX-2-matured DC showed higher cytotoxicity as compared to CTL generated with conventional mDC. Taken together, IRX-2-matured DC were more effective in inducing tumor cell-specific CTL *in vitro* as compared to conventional mDC.

### IRX-2 Matured DC Cross-present Antigen more Efficiently than Conventionally-matured DC

**Figure 4:**
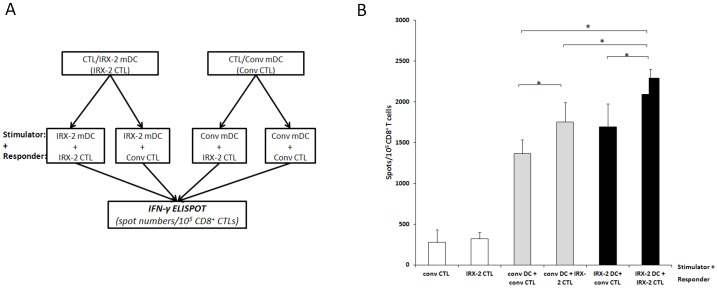
Cross-presentation of lysed tumor cells by mDC. CTL were generated using either IRX-2- or conventionally-matured mDC (IRX-2 CTL or conv CTL) as described in Materials and Methods. ELISPOT assays were performed using either IRX-2- or conventionally-matured mDC loaded with lysed PCI13 and co-incubated for 24 h with IRX-2 CTL or conv CTL. The highest number of spots was observed when IRX-2 mDC were co-incubated with IRX-2 CTL from the same donor (p<0.05 as compared to the other possible combinations of CTL and mDC). Co-incubation of IRX-2 mDC with conv CTL or conv mDC with IRX-2 CTL led to a similar number of spots, while co-incubation of conv CTL with conv mDC led to a significantly lower number of spots. IRX-2 and conv CTL showed similar levels of IFN-γ release in the absence of mDC (white bars). The mean number of spots per 10^5^ CD8^+^ T cells ± SEM from 4 independent experiments is shown.

Knowing that both conventionally- and IRX-2-matured DC are able to cross-prime PCI-13 specific CTL populations, we decided to use these *in vitro* generated CTL to explore the ability of mDC to cross-present tumor antigens. As summarized in Figure 4A, CTL were generated by IVS using mDC, which were either matured by conventional cytokines (Conv CTL) or IRX-2 (IRX-2 CTL). PCI-13 HNSCC cells were used as an antigen source for both types of CTL. DC matured either by IRX-2 or the conventional cytokines were loaded with a PCI-13 cell-lysate and tested for the presence of HLA-Class-I-peptide complexes on their surface. These surface complexes were recognized by autologous conventional CTL and IRX-2 CTL as shown in IFN-γ ELISPOT assays. Importantly, phagocytosis of lysed tumor cells was similar in both DC preparations (data not shown). Figure 4B shows that IRX-2 CTL showed a higher number of IFN-γ spots when co-incubated with IRX-2-matured DC than when incubated with conventional DC (p<0.05). The data suggest that IRX-2-matured DC are able to cross-present antigens derived from PCI-13 cells more efficiently than those matured with a conventional cytokine mixture.

## Discussion

IRX-2, a novel multi-component biologic, has been used for therapy of patients with HNSCC in a phase II clinical trial [13]. The therapy consisted of perilymphatically-delivered IRX-2 in combination with low-dose cyclophosphamide and a cyclooxygenase inhibitor, indomethacin, as well as zinc in a multivitamin formulation [14]. This regimen, administered prior to surgery, was shown to increase lymphocyte infiltration into the tumor and T-cell activation in situ as compared to biopsy tissue obtained prior to treatment [13]. It also induced relatively minor but significant changes in the peripheral blood lymphocyte subsets [16]. Further, overall survival (OS) was shown to be significantly improved in the patients whose tumors were infiltrated with T cells [13]. In view of this *in vivo* evidence for mobilization and activation of T-cells by IRX-2, and their correlation with improved OS, we considered the possibility that IRX-2 enhanced TA processing and presentation by DC, thereby resulting in more effective anti-tumor immunity. We tested this hypothesis using DC derived from monocytes of HNSCC patients and, specifically, evaluating IRX-2 effects on the expression of the APM components and on TA presentation to T cells. It is well documented that only adequately matured DC are able to cross-prime T cells and that only mDC migrate to lymph nodes where they can prime T-cell responses [17], [18]. Further, mDC produce higher levels of IL12p70, the cytokine necessary for Th1 and CTL responses, than iDC [15], [17], [19]. In addition, maturation greatly enhances antigen presentation and APM component expression as well as the expression of co-stimulatory molecules on the surface of DC [10], [17], [20]. Therefore, optimal maturation is essential DC function.

Various combinations of cytokines and/or toll-like-receptor (TLR) ligands have been used to mature human monocyte-derived DC [17], [21]. Usually, these cocktails contain variable levels of multiple cytokines, e.g., 50 ng/ml TNF-α, 25 ng/ml IL-1β and 10 ng/ml IL-6 in the most widely used conventional cocktail (“the conv. mix”). In contrast, IRX-2 used in this study contained 15–60 times lower concentrations of these cytokines (Table S2) than the conv. mix. In direct comparisons, while the conv. mix induced higher expression levels of CD80, CD83 and CD86 on moDC, IRX-2 induced considerably higher expression levels of CCR7, CD11c and CD40. Importantly, IRX-2-matured DC not only expressed higher surface levels of CCR7 but also migrated better in response to the CCR7 ligand, CCL21. CCR7 is essential for DC migration to lymph nodes, where interaction of DC and T cells takes place [22], suggesting that IRX-2-matured DC might also migrate more efficiently *in vivo*. CD40, a member of the TNFR superfamily, serves as a receptor on DC which interacts with CD154 on CD4^+^ T cells, leading to further DC maturation, and it induces IL-12p70 production [23]. It has been suggested that DC expressing low levels of CD40 might induce tolerance instead of immunity [24]. The induction of high levels of CD40 on DC matured with IRX-2 indicates that these cells are unlikely to induce tolerance, a clearly undesirable event in cancer patients receiving immunotherapy. The function of CD11c, also known as complement rector 4, remains less clear, but studies indicate its role in cell adhesion but also in antigen presentation by DC [25]. A higher CD11c expression on IRX-2-matured DC than on conventionally-matured DC could thereby contribute to their superior antigen presentation and induction of CTL.

The APM expression in DC is necessary not only for the presentation of antigens derived from self-proteins via the classical cytosolic pathway but also for the effective cross-presentation of exogenous antigens in the context of MHC class I molecules [8]. In patients with cancer, the APM component expression is compromised, and its’ up-regulation is, therefore, desirable [10]. Remarkably, IRX-2 was found to be able to induce higher levels of APM expression than the conv. mix. It has been reported that cytokine mixtures containing INF-γ are especially efficient in up-regulating the APM component expression [9]. In contrast to the conv. mix, IRX-2 contains INF-γ which could explain the higher levels of LMP2, TAP1, TAP2 and Tapasin expression in mDC. On the other hand, IFN-γ alone is not a sufficient maturation signal for moDCs and only in combination with TLR or CD40 ligation enhances CCR7-driven DC migration and cytokine production [18]. Since IRX-2 up-regulated DC migration and IL-12p70 production, it is likely that a synergistic effect of INF-γ and other cytokines included in IRX-2 was responsible for the observed effects.

Recently, Lopez-Albeitero et al reported that cross-presentation of the MAGE3_271-279_ peptide correlated with TAP1 and TAP2 expression in APC in that higher expression of these APM components resulted in more effective presentation of the peptide to T cells [9]. In addition, it has been shown, that a higher density of MHC-class-I-peptide complexes on the surface of APC leads to more effective induction and expansion of the peptide-specific CTL [26]. We hypothesized, that DC matured in the presence of IRX-2 have a higher density of non-self-peptide-MHC Class I complexes on their surface and thus are more efficient in loading, transporting and presentation of these peptides. Indeed, using tumor-reactive CTL generated via IVS with PCI-13-loaded DC we showed that IRX-2 matured DC induced high-potency CTL. Although we found higher levels of the co-stimulatory molecules CD80 and CD86 on conventionally-matured DC, CTL generated in IVS cultures with IRX-2-matured DC turned out to be more effective in killing PCI-13 targets which served as an antigen source for cross-priming. It also appears that CTL generated in IVS with IRX-2-matured mDC, which have enhanced cross-priming capabilities, are more responsive to tumor-derived antigens in ELISPOT assays. These CTL gave the highest number of IFN-γ spots upon co-incubation with IRX-2-matured DC presenting the antigen. We, therefore, suggest that the superior cross-priming capacity of IRX-2 matured DC is due to better cross-presentation of tumor cell-derived antigens likely resulting from up-regulated expression of APM components. In turn, this suggests that APM plays the central role in regulating the density of tumor-derived peptides present on the surface of mDC and that this step is of critical importance in the preparation of DC-based anti-cancer vaccines. However, effective cross-priming of T cells by APC is also critically dependant on cytokine-mediated signaling (i.e., signal 3) [27]. IL-12p70 appears to be essential for CTL priming by DC [19], [28]. Okada et al. recently reported that clinical responses to DC-based vaccines correlated with IL-12p70 production by the DC used for therapy [29]. In contrast, IL-10, which is considered to be an inhibitory cytokine, has negative effects on priming of T-cell responses [30]. A higher ratio of IL-12p70/IL-10 in supernatants of IRX-2-matured DC suggests that these DC are more likely to prime CTL responses.

Since IRX-2 clearly increases the *in vitro* potency of moDC obtained from the peripheral circulation of cancer patients and might also do so *in vivo* [13], it appears to be a promising component of future DC-based anti-tumor vaccines. For vaccine production, its ability to enhance IL-12p70 production, migratory response to CCL-21, APM component expression and cross-presentation of tumor antigens to T cells by DC are especially important. In immunotherapy of cancer, IRX-2 delivery alone or together with DC-based vaccines could be considered in future randomized clinical trials to improve the efficacy of currently available treatments.

## Materials and Methods

### Blood Samples

Peripheral blood was obtained from 18 HNSCC patients and 12 age and sex matched HD. Blood was drawn prior to therapy. All subjects signed an informed consent approved by the Institutional Review Board of the University of Pittsburgh (IRB#991206). Patients were seen at the Outpatient Otolaryngology Clinic between May 2010 and May 2011. Clinicopathological characteristics are listed in Table 2.

### IRX-2

IRX-2 is a primary cell-derived biologic containing multiple well-defined cytokines and produced by stimulation of human peripheral blood mononuclear cells (PBMC) with phytohemagglutinin (PHA). IRX-2 production under cGMP has been described previously [31]. The IRX-2 lot used for all the described experiments below contains several cytokines at the concentrations shown in Table S1.

### Antibodies

The following flourochrome-labeled monoclonal antibodies (mAbs) purchased from Beckman Coulter (Brea, CA) were used: anti-CD3-ECD, anti-CD8-FITC, anti-HLA-DR-FITC and PeCy5, anti-CD3-FITC, anti-CD14-PeCy5, anti-CD11c-PeCy5, anti-CD40-PE, anti-CD80-FITC, anti-CD83-FITC and PeCy5, anti-CD86-PE. Anti-CCR7-FITC mAb was from R&D Systems (Minneapolis, MN). Appropriate isotype controls were purchased from Beckman Coulter and Beckton Dickinson.

The LMP2-specific mAb SY-1, the TAP1-specific mAb NOB-1, the TAP2-specific mAb NOB-2, the calreticulin-specific mAb TO-11 and the tapasin-specific mAb TO-3 were developed and characterized as described [32]–[34]. mAb were purified from ascitic fluid by sequential precipitation with ammonium sulphate and caprylic acid [35]. The purity of mAb preparations was assessed by SDS-PAGE. The activity of the mAb preparations was monitored by testing with a lymphoid cell lysate in Western blotting. Anti-HLA-A2 mAb, BB7.2 was used to determine HLA-A2 expression [36]. The secondary Ab used in an indirect staining procedure, FITC-conjugated goat anti-mouse IgG Abs were purchased from Caltag Laboratories (Burlingame, CA). An anti-human-MHC-Class I mAb (w6/32) was described previously [37].

### Cell Lines

The HLA-A2^+^ head and neck squamous cell carcinoma (HNSCC) cell line, PCI-13, generated in our laboratory as previously described [38], and the HLA-A2^+^ breast cancer cell line MCF-7 were cultured in plastic culture flasks (Costar, Cambridge, CA) under standard conditions (37°C, 5% CO_2_ in air) using RPMI1640 medium (Lonza, Walkersville, MD) supplemented with 10% (v/v) FBS (Gibco-Invitrogen, Carlbad, CA). Cell cultures were tested every 3 months for endotoxin and Mycoplasma and were found to be negative.

### Surface and Intracellular Staining for Flow Cytometry

Cells were incubated for 20 min on ice with human Fc-Block (eBioscience, San Diego, CA) according to the manufacturer’s instructions. Without washing, cells were stained as described previously [23].

All antibodies were pre-titrated on freshly-harvested and activated PBMC to determine optimal working dilutions.

Surface and intracellular staining of the various APM components was performed as previously described [10]. Samples were tested using a 4-color Beckman Coulter XL, and data were analyzed using the Expo32-Software.

### Isolation of PBMC and DC Culture

PBMC were isolated by centrifugation on Ficoll-Hypaque (GE Healthcare, Uppsala, Sweden) from heparinized venous blood drawn from HD or HNSCC patients. moDC were generated as described previously [15]. Briefly, after isolation, 5–10×10^6^ PBMC were seeded onto 6-well-plates (Beckton Dickinson, Franklin Lakes, NJ) in AIM V medium (Gibco-Invitrogen, Carlsbad, CA) and incubated for 2 h. A small portion of PBMCs was used for HLA-A2-typing by flow cytometry. Non-adherent cells were removed and cryoperserved. Adherent cells were resuspended in RPMI1640 medium (Lonza) containing 1000 IU/ml GM-CSF (Bayer, Seattle, WA), 1000 IU/ml IL-4 (Cellgenix, Freiburg, Germany) and 10% (v/v) FBS (Gibco-Invitrogen) and were cultured for 5 days. On day 5, immature DC (iDC) were either harvested and tested or used for maturation. To mature iDC, IRX-2 or a conventional maturation cocktail [39] containing TNF-α (50 ng/ml), IL-1β (25 ng/ml) and IL-6 (10 ng/ml) (all cytokines from Cellgenix) diluted in *ex vivo* 10 medium (Lonza) were added. Following 48 h incubation, mature DC (mDC) were harvested and used for phenotypic and functional studies.

### IL-12p70 and IL-10 Luminex^©^

IL-12p70 and IL-10 concentrations in cell supernatants were determined by using commercially available Luminex^©^ kits according to the manufacturer’s instructions (Invitrogen). IL-12p70 and IL-10 concentrations were also measured in IRX-2 mixed 1∶1 with cell culture medium to determine background cytokine levels. Background cytokine levels were then subtracted from experimental values.

### In vitro Migration Assay

DC migration was investigated as previously described [21]. Briefly, the lower chamber of 24 trans-well plates (Corning Inc., Corning, NY) with polycarbonate membranes and 5 µm pore size was filled with 200 µl of RPMI1640 media containing 10% FBS and CCL21 (Peprotech, Rocky Hill, NJ) used at concentrations ranging from 0 to 100 ng/ml. Next, mDC (1×10^5^/100 µl medium) were seeded in the upper chamber, and plates were incubated for 2 h at 37°C. Cells in the lower chamber were counted using a Z1 Beckman Coulter particle counter.

### In vitro Sensitization (IVS) of CD8^+^ T Cells

CTLs were induced as previously described [37]. Briefly, PCI-13 cell lysates of were generated by 5 cycles of rapid freezing and thawing. DCs from HLA-A2^+^ donors were pulsed with tumor cell lysates for the last 24 h of maturation. Based on the number of tumor cells before lysis, tumor cells were added to DC at a 3∶1 ratio. TA-pulsed mDC were then irradiated (3000 rad) and washed with PBS. Autologous CD8^+^ T cells were isolated from cryopreserved PBMC by negative selection using magnetic bead separations (Miltenyi, Auburn, CA) and added to the mDC at the 10∶1 ratio. Cells were cultured in an atmosphere of 5% CO_2_ in air at 37°C for 7 days in AIM-V media containing 10 ng/ml IL-7 and 10 ng/ml IL-21 (Peprotech) and 5% (v/v) FBS. On day 7, fresh, tumor cell lysate-pulsed and irradiated mDC were added and T cells were cultured for additional 7 days in AIM V containing 20 IU/ml IL-2 (Peprotech), 5 ng/ml IL-7, 10 ng/ml IL-21 and 5% (v/v) FBS. On day 14, cells were harvested and used for functional studies.

### Phagocytosis Assay

Phagocytosis of lysed tumor cells by DC was determined using flow cytometry as previously described [40]. Briefly, PCI13 cells were stained with 2 µMol carboxyfluorescein succinimidyl ester (CFSE, Invitrogen) and extensively washed. Necrosis was induced in PCI13 cells by 5 cycles of freeze/thawing. iDC were stained with 4 µM PKH26 (Sigma-Aldrich) for 5 min at RT and washed afterwards. Maturation was induced by adding IRX-2 and the conventional maturation cocktail, each diluted 1∶1 with medium. Lysed, CFSE-labeled PCI-13 cells were added at the 1∶3 ratio after the first 24 h of maturation. Co cultures were harvested at different time points after the addition of the lysed tumor cells and phagocytosis was determined by flow cytometry by gating on PKH26^+^ events and expressed as the percentage of CFSE^+^ events within the PKH26 gate.

### Flow Cytometry Based Cytotoxicity Assay

CTL cytotoxicity was assessed by a modified flow cytometry based assay [41]. Briefly, PCI-13 and MCF-7 cells were stained with 2 µMol CFSE for 10 min at 37°C in the dark. Cells were washed and co-incubated with CTLs at various effector to target ratios for 4 h at 37°C. An aliquot of 1 µg/ml 7-amino-actinomycin D (7-AAD, Invitrogen) was then added to each tube, and the cells were incubated for an additional 20 min. Cells were acquired for analysis on a Beckman Coulter XL cytometer, detecting CFSE on FL1 and 7-AAD on FL4. Target cells were identified as CFSE-positive, and the percentage of 7-AAD positive target cells was determined. Target cells maintained for 4 h without CTL served as a negative control, and target cells incubated for 10 min at 56°C before a 4 h incubation served as a positive control for 7-AAD staining. The percentage of cytotoxic activity was calculated using the following formula: % specific lysis = 7-AAD^+^ targets minus spontaneous 7-AAD^+^ targets. MCF-7, a breast cancer cell line, was used as a specificity control in cytotoxicity assays. The HLA-class-I restriction of the cytotoxicity was tested by the preincubation of the target cells with 10 µg/ml of mAb W6/32 [37].

### IFN-γ ELISPOT Assay

IFN-γ ELISPOT assays were performed as previously described [23]. Briefly, wells of 96-well-plates with nitrocellulose membrane inserts were incubated with a capture anti-IFN-γ mAb (clone 1D1K, Mabtech, Nacka, Sweden) for 24 h at 4°C. Plates were washed, and the CTL (1×10^5^) generated in IVS assays as described above were added to each well followed by mDC (2.5×10^4^) pulsed with a tumor cell lysate. Plates were then incubated for 24 h at 37°C. Next, cells were removed by extensive washing, and a biotinylated secondary anti-IFN-γ Ab (clone 7-B6-1, Mabtech) was added for 2 h. After washing, plates were incubated with the avidin-peroxidase complex reagent, and aminoethylcabazole was added as a substrate. The reaction was terminated after 5 min, and spots were counted by computer-assisted image analysis (Zeiss ELISPOT 4.13.3, Jena, Germany). Background values (spots from wells containing mDC alone) were subtracted from experimental values (spots in wells containing mDC and CTL).

### Statistical Analysis

Data were analyzed using unpaired and paired students t tests. The p values <0.05 were considered significant.

## Supporting Information

Figure S1 **APM expression in iDCs from HD and HNSCC patients.** (**A**) Immature monocyte derived DCs generated from PBMC of HD (white bars) express significantly higher levels of TAP1 and TAP2 (*, p<0.01) than those generated from PBMC of HNSCC patients (black bars). Tapasin, Calreticullin and LMP2 expression was not significantly different in HNSCC patients and HD. The DC APM expression was determined by flow cytometry. The data are mean percentages ± SEM of cells positive for the indicated marker on cells obtained from 12 different HD and 12 HNSCC. (**B**) Representative histograms showing APM expression in iDC from HD and HNSCC patients. The shaded peaks represent isotype controls.
(JPG)

Table S1 Phenotype of iDC from healthy donors (HD) and HNSCC patients*.
(DOC)

Table S2 Concentrations of cytokines in the IRX-2 lot 051308 used for the described experiments*.
(DOC)
