# Supplementary figures and images for: IRX-2, a Novel Immunotherapeutic, Enhances Functions of Human Dendritic Cells
Source: PLoS One. 2013 Feb 7;8(2):e47234. doi: 10.1371/journal.pone.0047234 (PMC3567103; doi:10.1371/journal.pone.0047234)

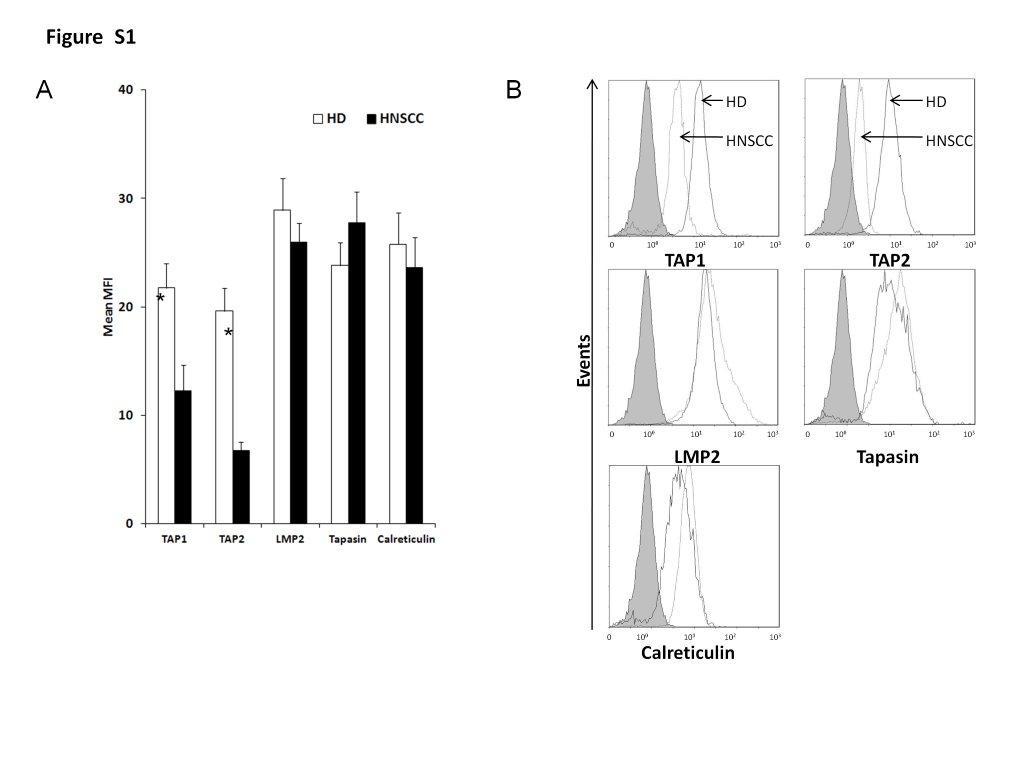

Supplement: Figure S1 — APM expression in iDCs from HD and HNSCC patients. (A) Immature monocyte derived DCs generated from PBMC of HD (white bars) express significantly higher levels of TAP1 and TAP2 (*, p<0.01) than those generated from PBMC of HNSCC patients (black bars). Tapasin, Calreticullin and LMP2 expression was not significantly different in HNSCC patients and HD. The DC APM expression was determined by flow cytometry. The data are mean percentages ± SEM of cells positive for the indicated marker on cells obtained from 12 different HD and 12 HNSCC. (B) Representative histograms showing APM expression in iDC from HD and HNSCC patients. The shaded peaks represent isotype controls. (JPG) [file pone.0047234.s001.jpg]
