# Supplementary material for: IRX-2, a Novel Immunotherapeutic, Enhances Functions of Human Dendritic Cells
Source: PLoS One. 2013 Feb 7;8(2):e47234. doi: 10.1371/journal.pone.0047234 (PMC3567103; doi:10.1371/journal.pone.0047234)
Supplement: Table S1 — Phenotype of iDC from healthy donors (HD) and HNSCC patients*. (DOC) [file pone.0047234.s002.doc]

**Table S1. Phenotype of iDC from healthy donors (HD) and HNSCC patients*.**

| Marker | HD (n=12) | |  | HNSCC patients (n=18) | |
| --- | --- | --- | --- | --- | --- |
|  | Mean % positive cells (±SEM) | Mean MFI (±SEM) |  | Mean % positive cells (±SEM) | Mean MFI (±SEM) |
| HLA-Class-I | 99±1 | 66±10 |  | 99±6 | 62±12 |
| HLA-DR | 91±10 | 5±2 |  | 90±7 | 4±1 |
| CD40 | 97±3 | 26±15 |  | 96±4 | 29±9 |
| CD11c | 95±3 | 19±9 |  | 93±4 | 19±9 |
| CD86 | 83±11 | 24±12 |  | 83±15 | 26±18 |
| CD80 | 33±15 | 4±1 |  | 33±23 | 5±1 |
| CD83 | 6±7 | 2±4 |  | 5±4 | 2±2 |
| CCR7 | 5±4 | 2±2 |  | 4±4 | 2±2 |
| CD14 | 4±2 | 1±2 |  | 5±3 | 1±1 |
| CD3 | 4±2 | 1±1 |  | 4±3 | 1±1 |

^*^ Phenotype of Immature monocyte derived DCs from HD and HNSCC patients as determined by flow cytometry.
