# Supplementary material for: IRX-2, a Novel Immunotherapeutic, Enhances Functions of Human Dendritic Cells
Source: PLoS One. 2013 Feb 7;8(2):e47234. doi: 10.1371/journal.pone.0047234 (PMC3567103; doi:10.1371/journal.pone.0047234)
Supplement: Table S2 — Concentrations of cytokines in the IRX-2 lot 051308 used for the described experiments*. (DOC) [file pone.0047234.s003.doc]

**Table S2. Concentrations of cytokines in the IRX-2 lot 051308 used for the described experiments*.**

| Cytokine | Concentration (ng/ml) |
| --- | --- |
| IL-2 | 5.0 |
| IL-1β | 0.94 |
| IFN-γ | 2.2 |
| TNF-α | 1.7 |
| IL-8 | 35.3 |
| IL-6 | 1.2 |
| GM-CSF | 0.27 |

^*^The IRX-2 lot was supplied by IRX Therapeutics Inc. (Farmingdale, NY). Cytokine levels were determined by ELISA and provided by IRX Therapeutics Inc.
